# Strength Development and Microstructural Observations of Concrete Incorporating Corn Straw Ash and KH570-Treated Crumb Rubber

**DOI:** 10.3390/polym18212614

**Published:** 2026-10-22

**Authors:** Kai Gao, Tengku Anita Raja Hussin, Qijing Xia, Jinyuan Zhang, Yi Shen, Vin Cent Tai

**Affiliations:** 1 Department of Building Engineering, Harbin Vocational University of Construction Technology, Harbin 150025, Chinashenyi20110510@163.com (Y.S.); 2 Centre for Infrastructure Geo-Hazards and Sustainability Materials, Faculty of Engineering, Built Environment and Information Technology, SEGi University, Kota Damansara, Petaling Jaya 47810, Selangor, Malaysiataivincent@segi.edu.my (V.C.T.); 3 College of Civil Engineering and Transportation, Northeast Forestry University, Harbin 150040, China

**Keywords:** corn straw ash, crumb rubber, KH570 surface treatment, mechanical performance, strength prediction

## Abstract

Strength losses and poor compatibility between recycled crumb rubber (CR) and cement paste constrain the use of waste-tire polymers in concrete. This study investigated the combined use of corn straw ash (CSA) and KH570-treated crumb rubber (CR) through mechanical testing, microstructural characterization, and compressive strength prediction. Fifteen mixtures were examined, with compressive strength measured at 3, 7, and 28 d and flexural strength at 28 d. Among the investigated CSA replacement levels at 10% CR, 8% CSA produced the highest 28 d compressive and flexural strengths of 39.28 and 7.1 MPa, respectively. These values were 15.1% and 20.3% higher than those of the corresponding rubberized concrete without CSA, and 15.2% and 16.4% higher than those of the untreated-rubber mixture. The combined treatment therefore partially compensated for rubber-induced strength losses. X-ray diffraction, scanning electron microscopy, and Fourier-transform infrared spectroscopy provided complementary evidence consistent with matrix refinement and improved rubber–paste interfacial compatibility. Backpropagation (BP), genetic-algorithm-optimized BP, and particle-swarm-optimized BP (PSO-BP) models were compared using 135 experimental records. PSO-BP yielded the lowest test-set root-mean-square error of 2.1713 MPa under the adopted data partition. These findings demonstrate the potential of combining agricultural ash incorporation with waste-rubber surface treatment to improve the strength performance of rubberized concrete and provide an experimental basis for composition selection.

## 1. Introduction

Cement is a principal raw material in concrete production. However, cement manufacturing is a carbon-intensive industry, emitting approximately 1.35 billion tonnes of carbon dioxide (CO_2_) annually and accounting for approximately 8% of total CO_2_ emissions [1,2]. Growing awareness of environmental protection and resource sustainability has encouraged the exploration of more sustainable construction strategies. In recent years, the use of industrial [3] or agricultural wastes [4,5] to replace conventional raw materials in concrete has attracted considerable attention. Crop straw and waste tires are widely available, and their improper stockpiling or burning creates environmental burdens. Processing these wastes for use in concrete can expand resource recovery pathways while reducing the consumption of cement or natural aggregates. Agricultural ash can modify the cementitious matrix, whereas crumb rubber introduces a deformable polymer phase and additional rubber–paste interfaces. Understanding how these changes influence mechanical performance is therefore important for developing concrete that accommodates both waste streams.

Northeast China is a major corn-producing region with abundant straw resources generated by corn cultivation [6]. Conventional disposal of surplus straw through burning or landfilling releases substantial greenhouse gases and causes severe environmental pollution [7]. Previous studies have incorporated straw powder directly into concrete; although this approach can improve deformation capacity, it may also reduce compressive strength [8]. Converting corn straw into corn straw ash (CSA) through appropriate thermal treatment therefore offers an important route to broader utilization and potential cement replacement. With suitable processing, reactive siliceous constituents in CSA can react pozzolanically with Ca(OH)_2_ produced during cement hydration to form additional hydration products. Its fine particles can also provide a filler effect, improving the cement matrix structure [9,10]. Shang et al. [11] further demonstrated the feasibility of converting corn straw into a supplementary cementitious material through material performance assessment and life cycle analysis. Lima et al. [12] prepared CSA using acid leaching, two-stage combustion, and grinding, and found that pretreatment improved its physicochemical characteristics and pozzolanic performance. These findings indicate that the effectiveness of CSA cannot be assessed solely from its source; the material characteristics resulting from processing must also be considered. Under suitable conditions, reactive constituents can participate in secondary reactions, while fine particles may contribute to pore filling. However, increasing cement replacement also intensifies the dilution effect associated with reduced clinker content. The influence of CSA on strength development thus reflects the combined effects of reaction, filling, and cement replacement, and its suitable dosage must be determined for the specific material system.

Research on corn cob ash (CCA) provides a broader basis for understanding the utilization of corn-derived residues in concrete. Although CCA and CSA originate from different parts of the plant, both have been investigated as constituents of cementitious materials. Bheel et al. [13] examined CCA as a fine-aggregate replacement in concrete containing ground granulated blast-furnace slag, illustrating the possibility of combining agricultural residues with other supplementary constituents. Șerbănoiu et al. [14] compared the effects of CCA and sunflower stalk ash on concrete strength and durability, highlighting the importance of ash type in determining performance. Oluremi et al. [15] investigated nanosilica addition to improve the early strength development of CCA concrete, while Oriola et al. [16] considered both compressive strength and thermal properties. Ouedraogo et al. [17] explored pretreatment of corn cobs to improve the performance of the resulting ash, Olaniyi [18] investigated the strength development of binders incorporating rice husk and corn cob ashes, and Otieno et al. [19] examined CCA–silica-fume blends in concrete. Together, these studies identify processing, replacement level, curing age, and complementary constituents as important variables in the effective use of corn-derived ashes. They also provide a basis for investigating whether agricultural ash incorporation can benefit concrete containing recycled polymer particles.

Crumb rubber (CR) produced from waste tires has been widely investigated as a partial replacement for natural aggregate [5,20,21]. Its incorporation provides a reuse route for a crosslinked polymer that differs substantially from mineral aggregate in stiffness and surface characteristics. These differences affect stress transfer and the development of the rubber–paste interface, frequently resulting in reduced compressive and flexural strengths. Consequently, research has increasingly focused on mitigating strength losses through modification of the surrounding matrix and treatment of the rubber surface. For example, Shao et al. [22] investigated the combined effects of recycled tire rubber and carbon nanotubes on concrete mechanical properties and microstructure. Such approaches highlight the value of considering the matrix and the polymer–cement interface together when designing rubberized concrete. In a different composite system, Karagöz et al. [23] combined mechanical, thermal, and morphological characterization of walnut-shell- and nanoclay-filled high-density polyethylene. Although the matrix differs from cement paste, this work illustrates the value of relating filler–matrix interactions to macroscopic performance.

Silane coupling agents offer a means of modifying the interactions between rubber particles and cementitious materials. Their organic functional groups and hydrolyzable silane groups provide a basis for tailoring compatibility between organic and inorganic constituents. KH570 contains a methacryloxy functional group, whereas KH550 is amino-functional. These different organic functionalities provide a basis for examining their interactions with rubber and cementitious phases. Yu et al. [24] reported favorable mechanical performance for KH570-treated rubber–cement composites relative to the other silanes tested, supporting the selection of KH570 for evaluation in the present CSA-containing matrix. In this context, a comparison between treated and untreated rubber can establish the mechanical response associated with the selected treatment, while complementary microstructural observations help interpret that response.

The combined incorporation of CSA and CR has also been investigated. Zhang et al. [25] studied concrete containing CSA and KH550-treated CR and reported that suitable CSA incorporation could mitigate rubber-related strength losses. Ji and Xia [8] investigated KH570-treated rubber in concrete containing unburned straw powder, establishing a related route for combining rubber surface treatment with agricultural constituents. Recent studies have further extended agricultural ash–rubber combinations to the flexural and shear responses of reinforced concrete beams containing rice husk ash (RHA) and CR [26,27]. In parallel, Han et al. [28] investigated KH570 pretreatment for the flexural-fatigue behavior of rubberized concrete. Table 1 compares these studies with the present work in terms of materials, modification approaches, and research scope.

CSA, corn straw ash; CR, crumb rubber; RHA, rice husk ash; CNT, carbon nanotube; XRD, X-ray diffraction; SEM, scanning electron microscopy; FTIR, Fourier-transform infrared spectroscopy; TGA, thermogravimetric analysis; BP, backpropagation neural network; GA, genetic algorithm; PSO, particle swarm optimization.

Alongside experimental evaluation, data-driven modeling can assist in describing relationships between mixture composition, curing age, and compressive strength. Backpropagation (BP) neural networks provide a nonlinear mapping between these variables, while genetic algorithms (GA) and particle swarm optimization (PSO) can be used to optimize network parameters. Xia and Ji [29] applied optimized neural networks to rubber–straw concrete, demonstrating the relevance of these approaches to concrete containing multiple waste-derived constituents. Extending this comparison to CSA–CR concrete provides a complementary means of assessing the composition–strength relationships within the experimental dataset.

Accordingly, this study investigates concrete incorporating CSA and KH570-treated CR through an integrated program of mechanical testing, microstructural characterization, and strength modeling. Compressive strength is evaluated at 3, 7, and 28 d, and flexural strength at 28 d, across the selected CSA and CR replacement levels. An untreated-rubber mixture is included to assess the effect of KH570 treatment at the same CSA and CR contents. X-ray diffraction, scanning electron microscopy, and Fourier-transform infrared spectroscopy are used to examine phase, morphological, and spectral characteristics associated with the measured strength responses. BP, GA-BP, and PSO-BP models are also compared for compressive strength prediction. The contribution of this study lies in linking dosage-dependent strength development and the treated–untreated comparison with complementary microstructural evidence in the CSA–KH570-treated CR system, providing an experimental basis for the combined utilization of agricultural ash and recycled tire rubber in concrete.

## 2. Materials and Methods

### 2.1. Raw Materials

**Figure 1:**
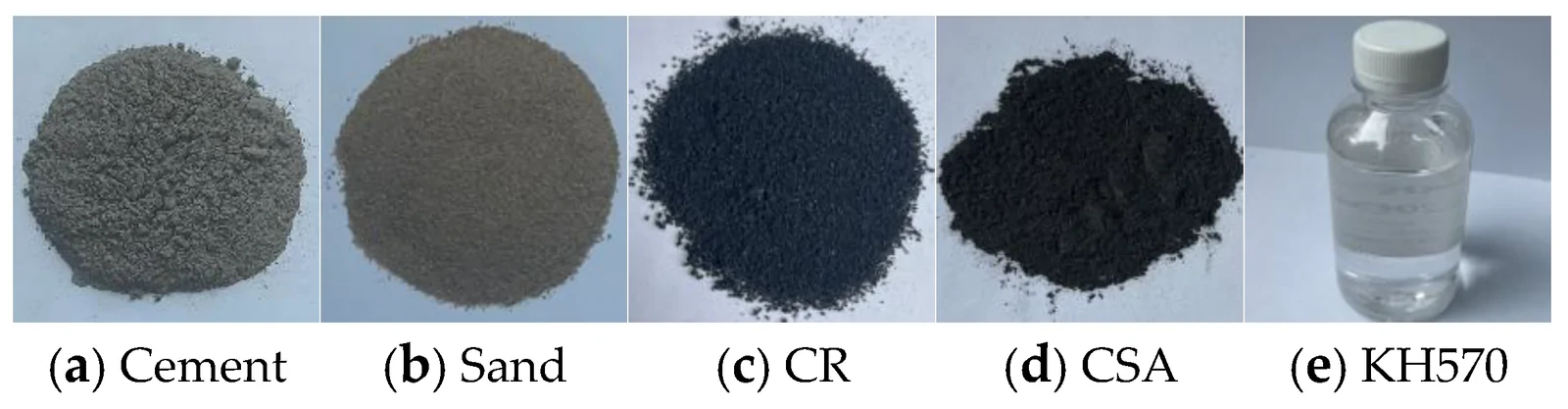
Raw materials used in this study.

The raw materials are shown in Figure 1. P·O 42.5 ordinary Portland cement was obtained from a cement plant in Harbin, Heilongjiang Province, China. Natural river sand from Harbin was used as the fine aggregate; it was medium sand with a fineness modulus of 2.3 and a bulk density of 2238 kg/m^3^. Well-graded crushed stone with a particle size of 5–20 mm was supplied by a building materials company in Harbin, China and used as the coarse aggregate. Crumb rubber (CR), with a particle size of 20 mesh (approximately 0.85 mm sieve opening), a bulk density of 750 kg/m^3^, and a specific gravity of 0.84, was supplied by Dujiangyan Huayi Rubber Products (Dujiangyan, China) and used to partially replace the sand. Tap water was used for specimen preparation and curing. A polycarboxylate-based high-range water-reducing admixture with a water reduction rate of approximately 28.5% was used to improve concrete workability. The silane coupling agent KH570, supplied by Kangjin Chemical Products Co., Ltd. (Guangzhou, China), was used to improve the compatibility of CR with the other concrete constituents. Corn straw for CSA production was collected from local farmland in Harbin, Heilongjiang Province. The straw was first burned at approximately 280 °C for 2 h and then air-cooled. The resulting ash was subsequently calcined at 500 °C for 2 h.

### 2.2. Mix Design and Experimental Procedures

#### 2.2.1. Mix Design of Concrete

This study aimed to use the solid wastes CSA and CR to produce sustainable concrete. Fifteen mixtures incorporating CSA and CR were prepared in accordance with the Chinese Specification for Mix Proportion Design of Ordinary Concrete (JGJ 55–2011) [30] to investigate concrete mechanical properties. The mix proportions are listed in Table 2. Plain concrete (PC) was designed to meet strength class C40, with a water-to-cement ratio of 0.4. CR replaced fine aggregate on an equal-volume basis at replacement levels of 5%, 10%, and 15%. CSA was incorporated at 0%, 6%, 8%, and 10% by mass of cement as a pozzolanic material and microfiller to partially replace cement. Mixtures were designated CαRβ, where α denotes the CSA mass replacement percentage and β denotes the CR volume replacement percentage. For example, C6R10 denotes a mixture with 6% cement replacement by CSA and 10% fine aggregate replacement by CR. C8UR10 denotes a mixture with 8% cement replacement by CSA and 10% sand replacement by untreated CR.

#### 2.2.2. Pretreatment of CR

**Figure 2:**
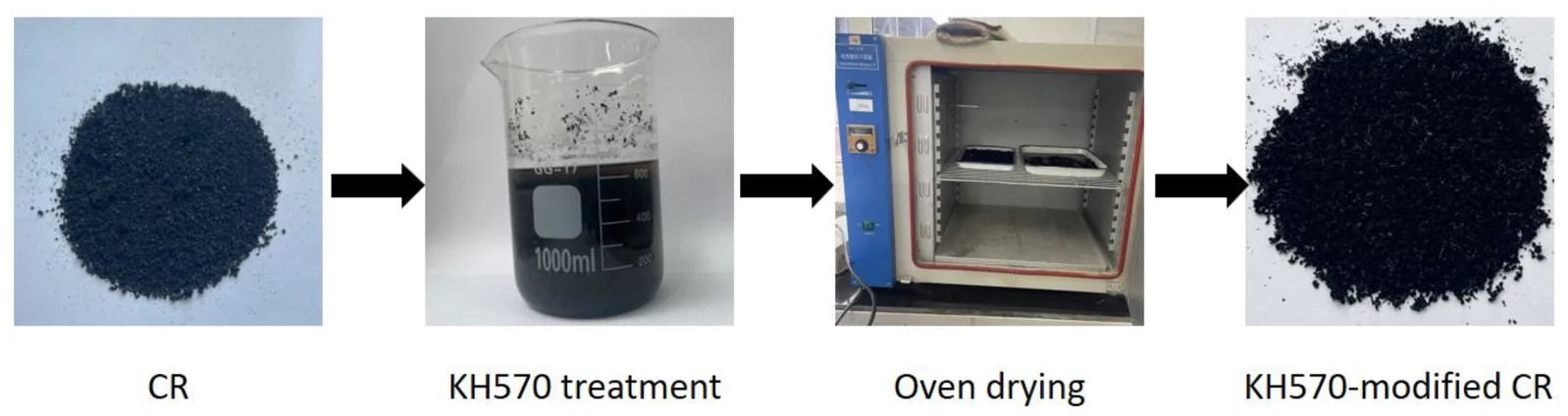
KH570 treatment procedure for CR.

Rubber is hydrophobic and therefore bonds poorly with cement. To improve adhesion between CR particles and cement, CR was treated with the silane coupling agent KH570. The dosage of KH570 was fixed at 2% by mass of CR for all modified rubber mixtures. KH570 was first added to a 95% aqueous ethanol solution and stirred at 20 °C for 10 min. CR was then introduced, and stirring continued for another 10 min. The mixture was subsequently heated to 75 °C at 1.5 °C/min and stirred for an additional 10 min. Finally, the treated CR was oven-dried at 110 °C for subsequent use [17]. The treatment procedure is illustrated in Figure 2.

#### 2.2.3. Specimen Preparation

The concrete specimens were prepared through mixing, casting, and curing. First, the aggregate and sand were dry-mixed for 1 min, followed by the addition of cement, CSA, and pretreated CR, with further mixing for 1 min. This sequence was adopted to improve the dispersion of the lightweight CR particles and enhance their interfacial bonding with the cementitious matrix. Subsequently, half of the mixing water containing the dissolved water reducer was added and mixed for 1 min. The remaining water was then introduced, followed by another 1.5 min of mixing. Before casting, the inner surfaces of the molds were coated with release oil. The fresh concrete was placed into the molds and mechanically vibrated for 15 s. Excessive vibration was avoided to minimize the upward migration of lightweight CR particles and maintain a uniform distribution within the concrete. After 24 h, the specimens were demolded and transferred to a curing chamber for standard curing in accordance with GB/T 50081-2019 [31].

**Figure 3:**
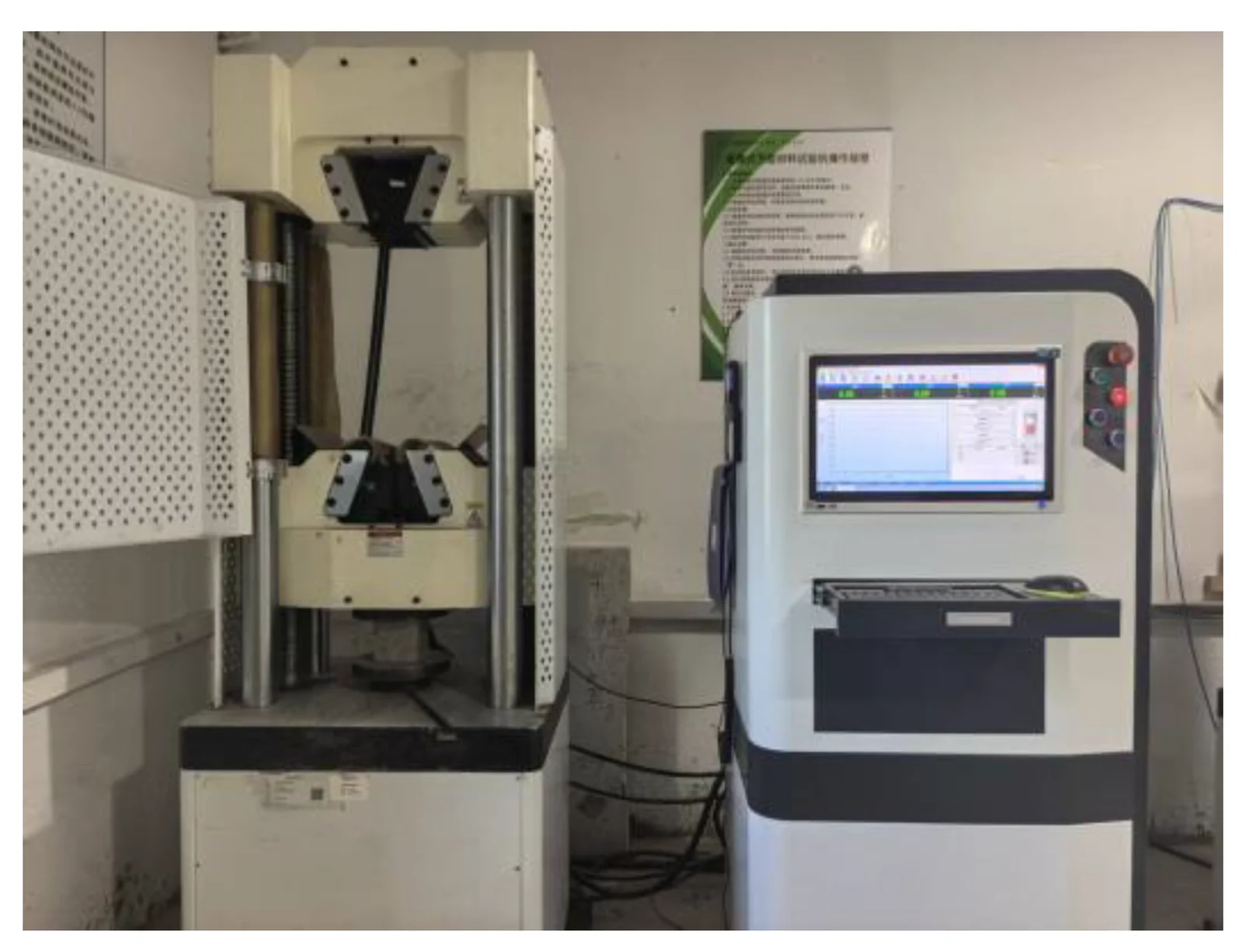
Testing equipment.

According to the Chinese standard GB/T 50081-2019 [31], compressive strength tests were conducted on 100 mm × 100 mm × 100 mm cubic specimens at 3, 7, and 28 d, while flexural strength tests were performed on 100 mm × 100 mm × 400 mm prismatic specimens at 28 d. The flexural strength was measured. The loading rate for flexural strength was (50 ± 10) N⋅s^−1^, and that for compressive strength was (2400 ± 200) N⋅s^−1^. All specimens were prepared using mechanical mixing and vibration compaction. Three replicate specimens were tested for each mixture at each designated testing age. The testing equipment is shown in Figure 3. A total of 180 specimens were tested, comprising 135 specimens for compressive strength and 45 specimens for flexural strength.

### 2.3. Microstructural Test

A Hitachi S-4800 field-emission scanning electron microscope (SEM) (Hitachi, Ltd., Tokyo, Japan) was used to examine the concrete microstructure and hydration product morphology. Representative fragments less than 1 cm in length and width and less than 0.5 cm in thickness were collected from the interiors of fractured specimens. Before testing, the samples were immersed in absolute ethanol to stop further hydration and then oven-dried at 50 °C for 12 h.

The crystalline phases and mineralogical composition were characterized using a Bruker D8 Advance X-ray diffractometer (Bruker Corporation, Billerica, MA, USA) with Cu Kα radiation. XRD patterns were collected over a 2θ range of 10–80° at a scanning rate of 2°/min. Before testing, the samples were dried for 48 h, finely ground, and passed through a 200-mesh sieve. Approximately 3 g of powder was used for each measurement.

Fourier-transform infrared spectroscopy was performed using a Thermo Scientific Nicolet i10 FTIR spectrometer (Thermo Fisher Scientific, Waltham, MA, USA) to identify characteristic functional groups and evaluate changes in the chemical environment of the investigated mixtures. Following hydration termination and drying, the concrete samples were ground into a homogeneous fine powder. The spectra were used to evaluate changes in the chemical bonding characteristics of hydration products and possible interfacial interactions induced by CSA and KH570-modified CR.

### 2.4. Development of Compressive Strength Prediction Models

**Figure 4:**
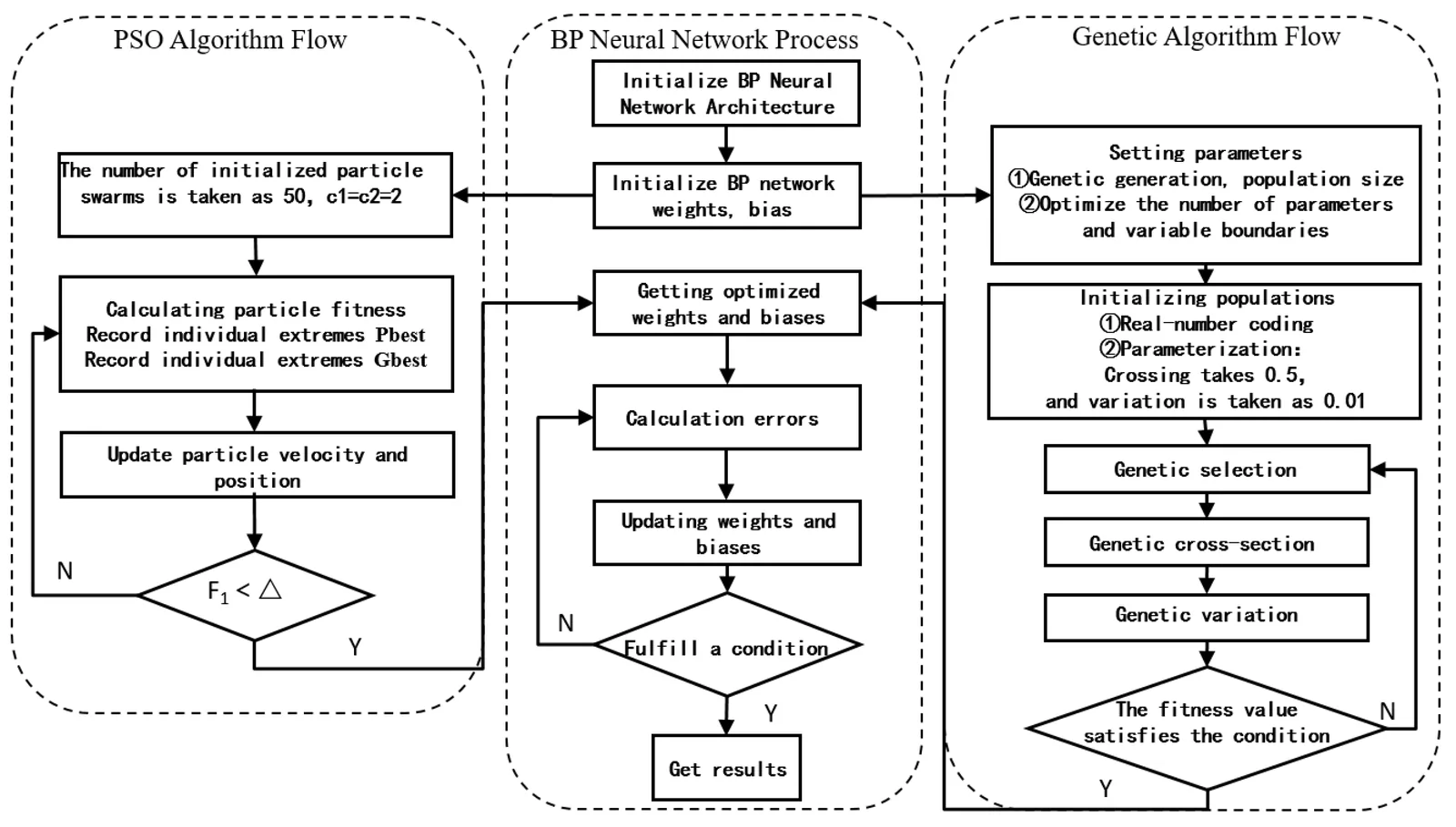
Development procedures for the GA-BP and PSO-BP neural networks.

To develop compressive strength prediction models for corn straw ash–rubber concrete (CCC), six input variables were selected from the experimental mixture designs: CSA content, CR content, sand content, silane coupling agent content, cement content, and curing age. Compressive strength was used as the output variable. A backpropagation (BP) neural network was employed to establish the nonlinear relationship between these inputs and compressive strength. Genetic algorithm (GA) and particle swarm optimization (PSO) were separately applied to optimize the initial network weights and biases, yielding the GA-BP and PSO-BP models, respectively. The optimization procedures are illustrated in Figure 4. The dataset comprised 135 compressive-strength observations obtained from 15 mixtures, three curing ages (3, 7, and 28 d), and three replicate specimens per mixture–age combination. In the original analysis, 95 observations were assigned to the training set and 40 to the test set through record-wise random partitioning. Because observations from the same mixture could appear in both subsets, the resulting performance was interpreted as prediction within the sampled mixture space rather than as evidence of generalization to unseen mixtures.

To assess prediction performance for mixtures excluded from model fitting, additional five-fold mixture-level cross-validation was conducted. Each fold comprised three complete mixtures for testing (27 observations), while the remaining twelve mixtures were used for training (108 observations). All curing ages and replicate specimens associated with a given mixture were assigned to the same fold. Identical fold assignments were used for all three models. Each model was initialized and trained independently in each fold, with GA and PSO optimization repeated using only the corresponding training data. Normalization parameters were estimated exclusively from the training data and subsequently applied to the held-out test data. The test data were not used for parameter optimization, early stopping, or model selection.

Model performance was evaluated using root mean square error (RMSE), mean absolute error (MAE), the coefficient of determination (R^2^), and Pearson’s correlation coefficient (R). RMSE and MAE quantify prediction errors in MPa, while R^2^ evaluates predictive performance relative to a baseline prediction equal to the mean observed strength in the evaluated subset. Pearson’s R measures the linear association between predicted and observed strengths. R^2^ was calculated directly from the prediction residuals rather than as the square of Pearson’s R. For mixture-level cross-validation, MAE, RMSE, and R^2^ were calculated separately for each held-out fold and reported as the mean ± sample standard deviation across the five folds.

## 3. Results and Discussion

### 3.1. Raw Material Characterization

**Figure 5:**
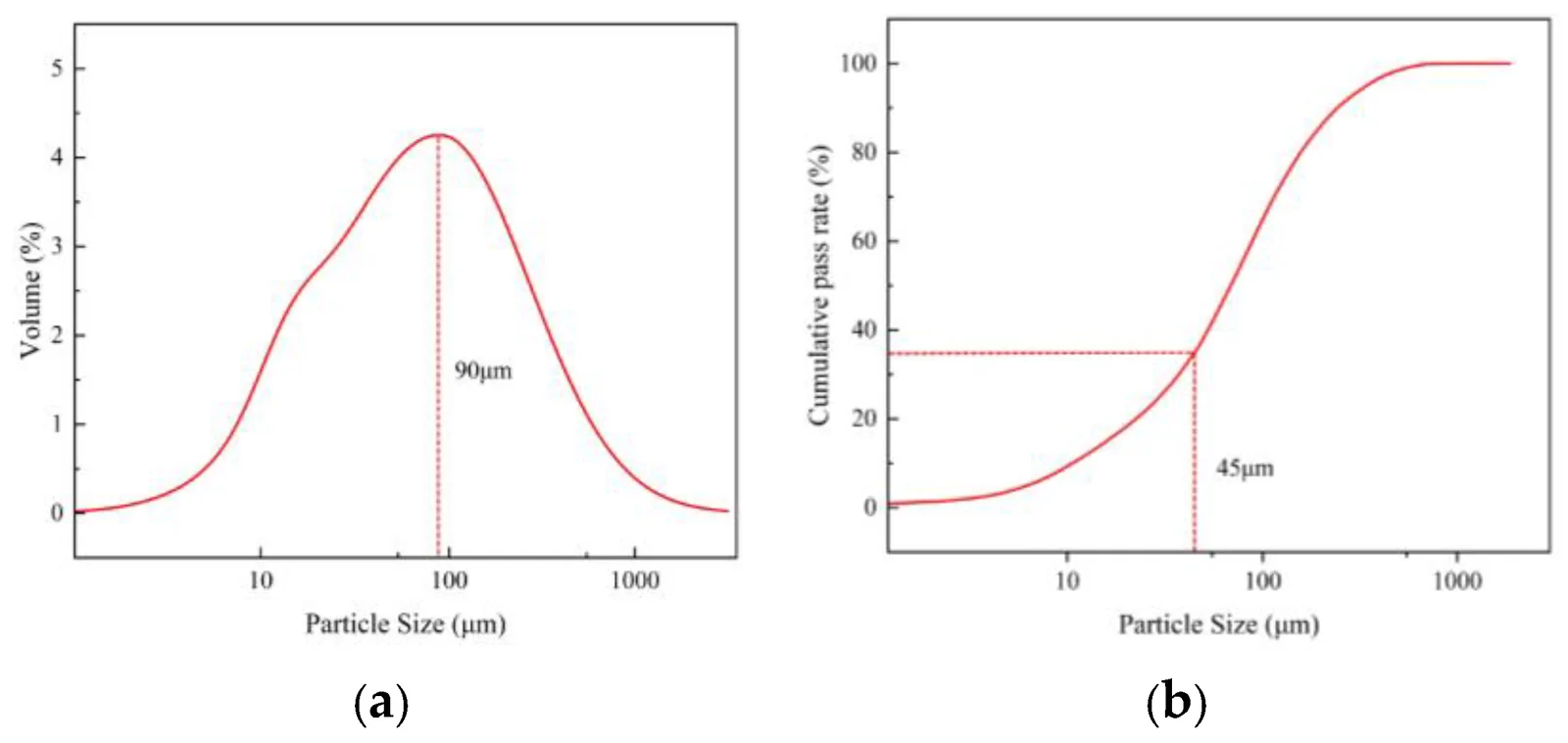
Particle sizes of the CSA sample: (**a**) Particle size distribution. (**b**) Cumulative passage rate.

Figure 5a,b show the particle-size distribution and cumulative passing curve of the CSA, respectively. As shown in Figure 5a, the CSA exhibited a relatively broad particle-size distribution, with the main particle-size peak occurring at approximately 90 μm. A weak shoulder was also observed in the finer-particle region, indicating that the CSA contained a certain proportion of fine particles in addition to the dominant coarser fraction. The broad distribution suggests that the CSA particles were not concentrated within a narrow size range. According to the cumulative passing curve in Figure 5b, approximately 35% of the CSA particles were smaller than 45 μm, corresponding to a 45 μm sieve residue of approximately 65%, which exceeds the threshold of 20% specified in the pozzolanic materials specification [32].

The chemical composition of CSA is summarized in Table 3. SiO_2_ was the predominant oxide, accounting for 59.42 wt.%, followed by Al_2_O_3_ (11.53 wt.%) and Fe_2_O_3_ (2.80 wt.%). The combined content of SiO_2_, Al_2_O_3_, and Fe_2_O_3_ was approximately 73.75 wt.%, indicating that the CSA was predominantly a silica–alumina-rich material. In addition, the CSA contained relatively small amounts of K_2_O, CaO, MgO, P_2_O_5_, SO_3_, and Na_2_O. The loss on ignition rate was 3.10%, which was lower than the allowable limit of 10% [33], indicating that during the combustion process, most of the residual organic matter and unburned carbon had been removed. The relatively high SiO_2_ content provides a compositional basis for potential pozzolanic reactivity.

**Figure 6:**
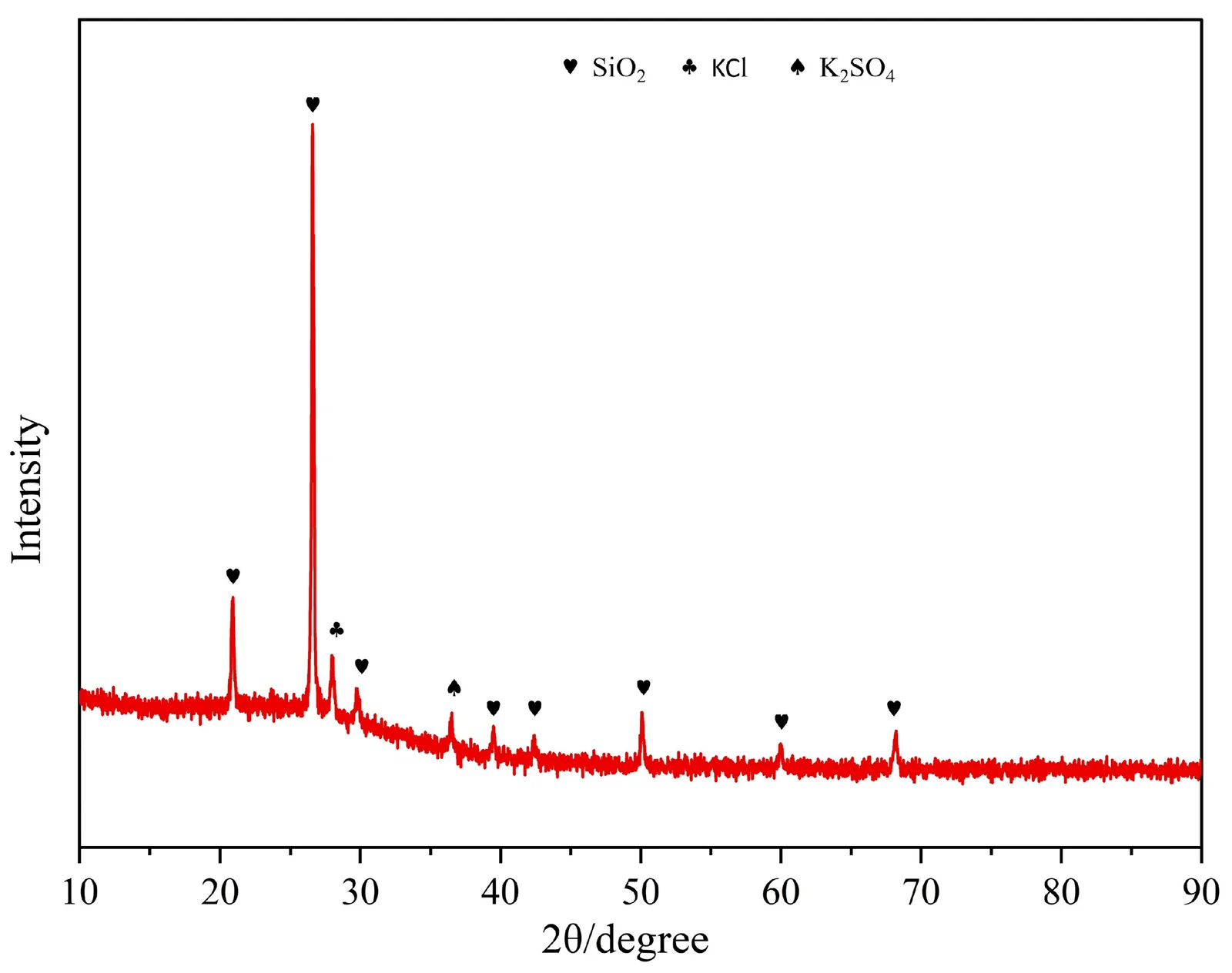
XRD spectra of CSA.

Figure 6 shows the XRD pattern of CSA. A pronounced diffraction peak was observed at approximately 2θ = 26.59°, which was mainly assigned to crystalline quartz (SiO_2_), together with several weaker crystalline diffraction peaks. Meanwhile, the elevated diffuse background over part of the diffraction range suggests the possible presence of an amorphous or poorly crystalline fraction. These results indicate that SiO_2_ in CSA is not entirely present in a highly reactive form, with a portion existing as relatively stable crystalline quartz. The potential pozzolanic contribution of CSA is therefore more likely associated with its amorphous siliceous/aluminosilicate fraction rather than the total SiO_2_ content determined by XRF.

Based on the above results, CSA can be considered to possess a certain potential for pozzolanic reactivity. However, its relatively low proportion of fine particles may limit the extent of this reaction. The actual contribution of CSA is further interpreted in conjunction with the mechanical and microstructural results presented in the following sections.

### 3.2. Compressive Strength

**Figure 7:**
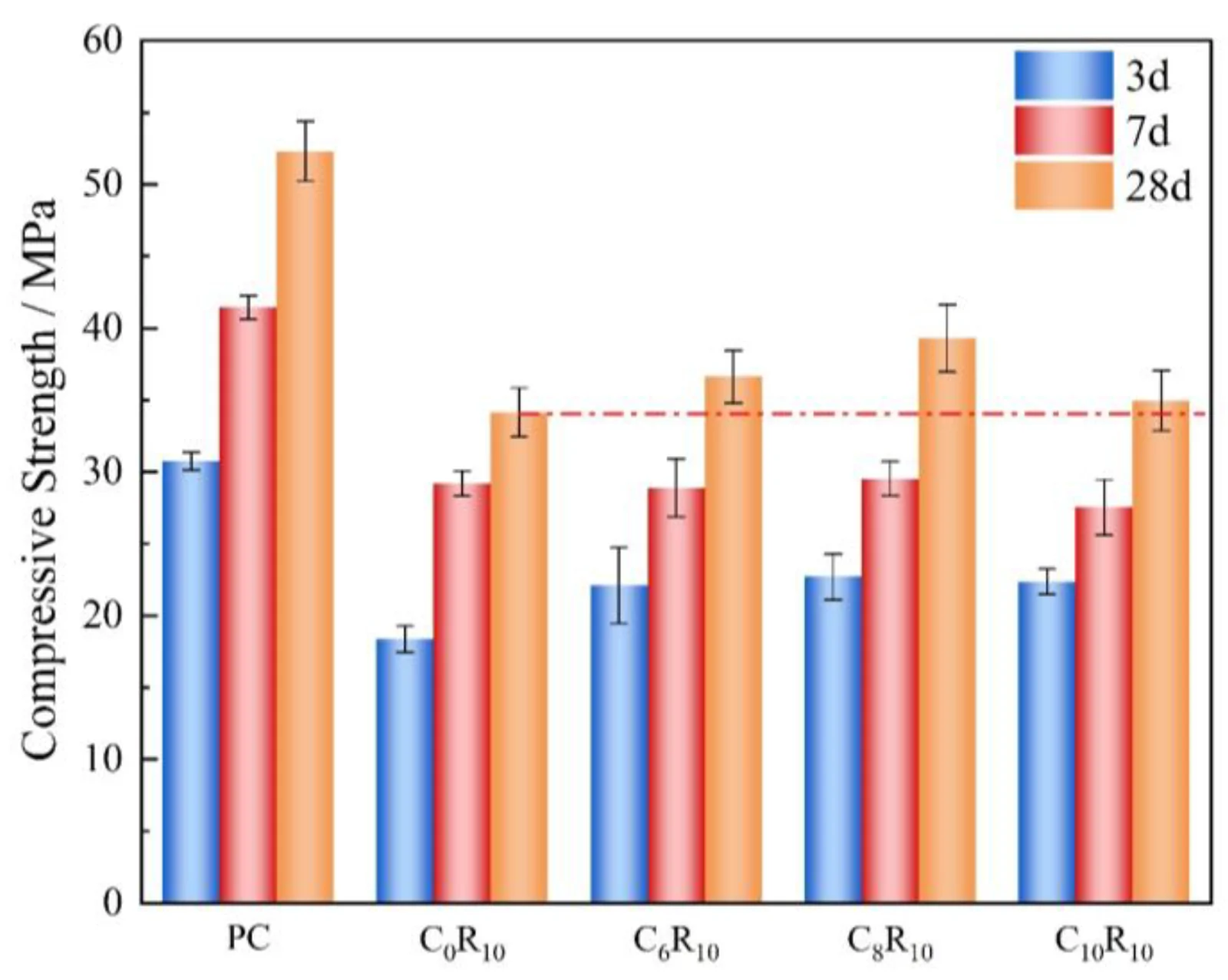
Compressive strength of concrete mixtures at different ages.

Figure 7 presents the compressive strength development of the concrete mixtures at 3, 7, and 28 d. The compressive strength increased with curing age for all mixtures. PC exhibited compressive strengths of 30.76, 41.43, and 52.31 MPa at 3, 7, and 28 d, respectively, whereas the corresponding values of C0R10 were 18.36, 29.18, and 34.13 MPa, representing reductions of 40.3%, 29.6%, and 34.8%. These results confirm that replacing natural sand with CR reduced compressive strength. This reduction can primarily be associated with the lower stiffness of rubber relative to natural aggregate and the relatively weak rubber–paste interfacial transition zone (ITZ), which may facilitate local deformation and stress concentration under compression.

At a constant CR replacement level of 10%, the incorporation of CSA produced a non-monotonic effect on compressive strength. The 28 d compressive strengths of C6R10, C8R10, and C10R10 were 36.61, 39.28, and 34.96 MPa, respectively, corresponding to increases of 7.3%, 15.1%, and 2.4% relative to C0R10. Among the investigated CSA contents, C8R10 exhibited the highest 28 d compressive strength. The improvement at moderate CSA contents may be attributed to a combination of particle-filling effects and the potential contribution of reactive amorphous siliceous/aluminosilicate constituents to secondary hydration reactions. This interpretation is consistent with the raw-material characterization and the XRD and SEM observations discussed in Section 3.1 and Section 3.4. However, increasing the CSA replacement level from 8% to 10% reduced the 28 d compressive strength by approximately 11.0%. The reduction at the higher replacement level may be associated with increased cement dilution and the relatively coarse particle-size distribution of the CSA, which could limit its effective participation in secondary reactions. Therefore, 8% CSA should be regarded as the best-performing dosage among the levels investigated in this study, rather than a universally optimal replacement level.

**Figure 8:**
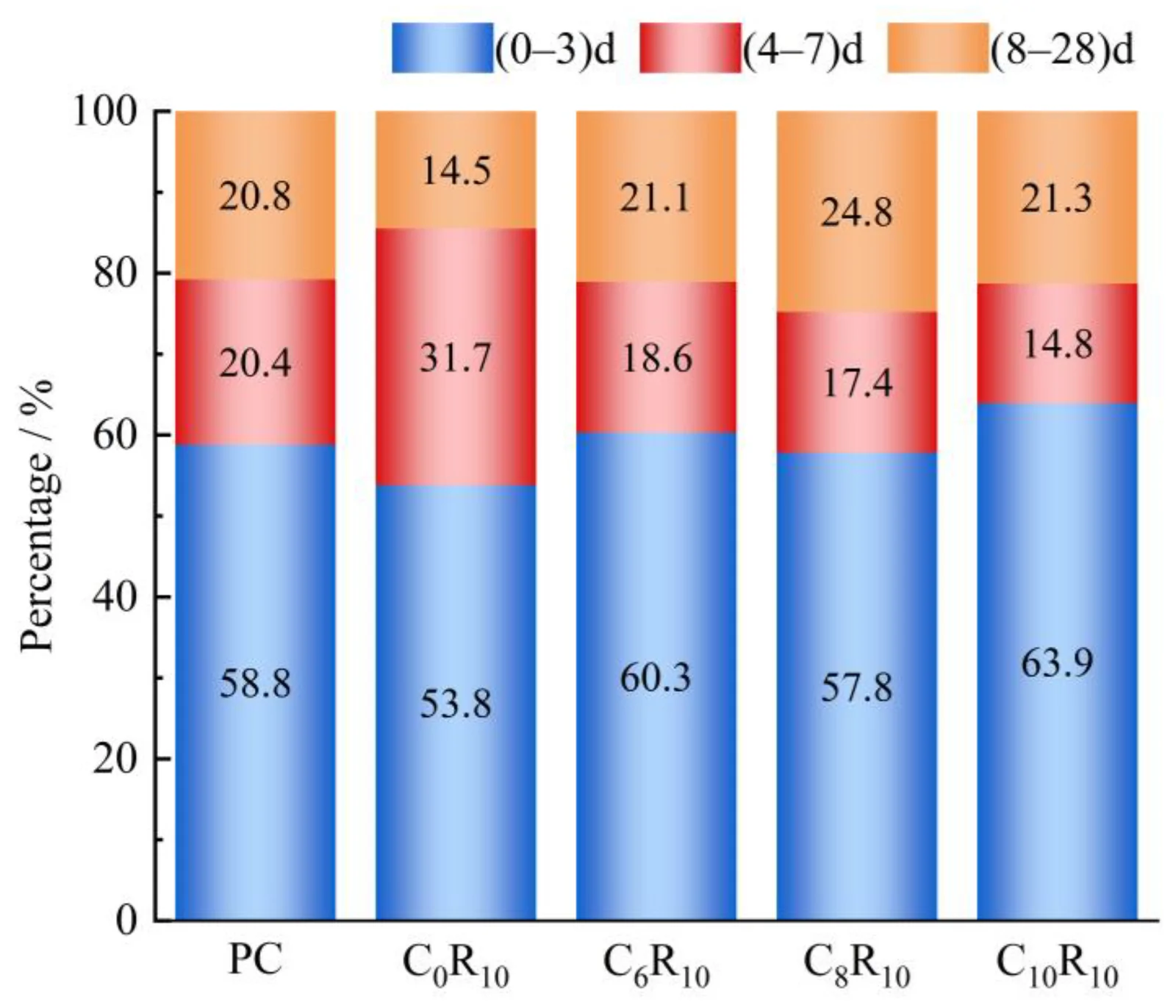
Contributions of different age intervals to the 28 d compressive strength.

Figure 8 further illustrates the contributions of different age intervals to the 28 d compressive strength. Strength developed during 0–3 d accounted for 53.8–63.9% of the 28 d strength, indicating that a major proportion of strength formed at early ages. For PC, the contributions of the 0–3, 4–7, and 8–28 d intervals were 58.8%, 20.4%, and 20.8%, respectively. C0R10 exhibited a contribution of 31.7% during 4–7 d, substantially higher than those of the other mixtures, but only 14.5% during 8–28 d. This indicates that rubber delayed early strength development while providing relatively limited potential for later strength gain. With CSA incorporation, the 8–28 d contributions reached 21.1% and 24.8% for C6R10 and C8R10, respectively, with C8R10 exhibiting the highest value. An appropriate CSA content thus improved strength development at intermediate and later ages. This may be associated with the relatively slow pozzolanic reaction of CSA: as curing proceeded, reactive siliceous constituents continued to consume CH and form secondary C–S–H gel, progressively filling capillary pores and improving the ITZ. In contrast, C10R10 developed 63.9% of its 28 d strength during 0–3 d but only 14.8% during 4–7 d, indicating that a higher CSA content did not produce a more favorable pattern of sustained strength development.

**Figure 9:**
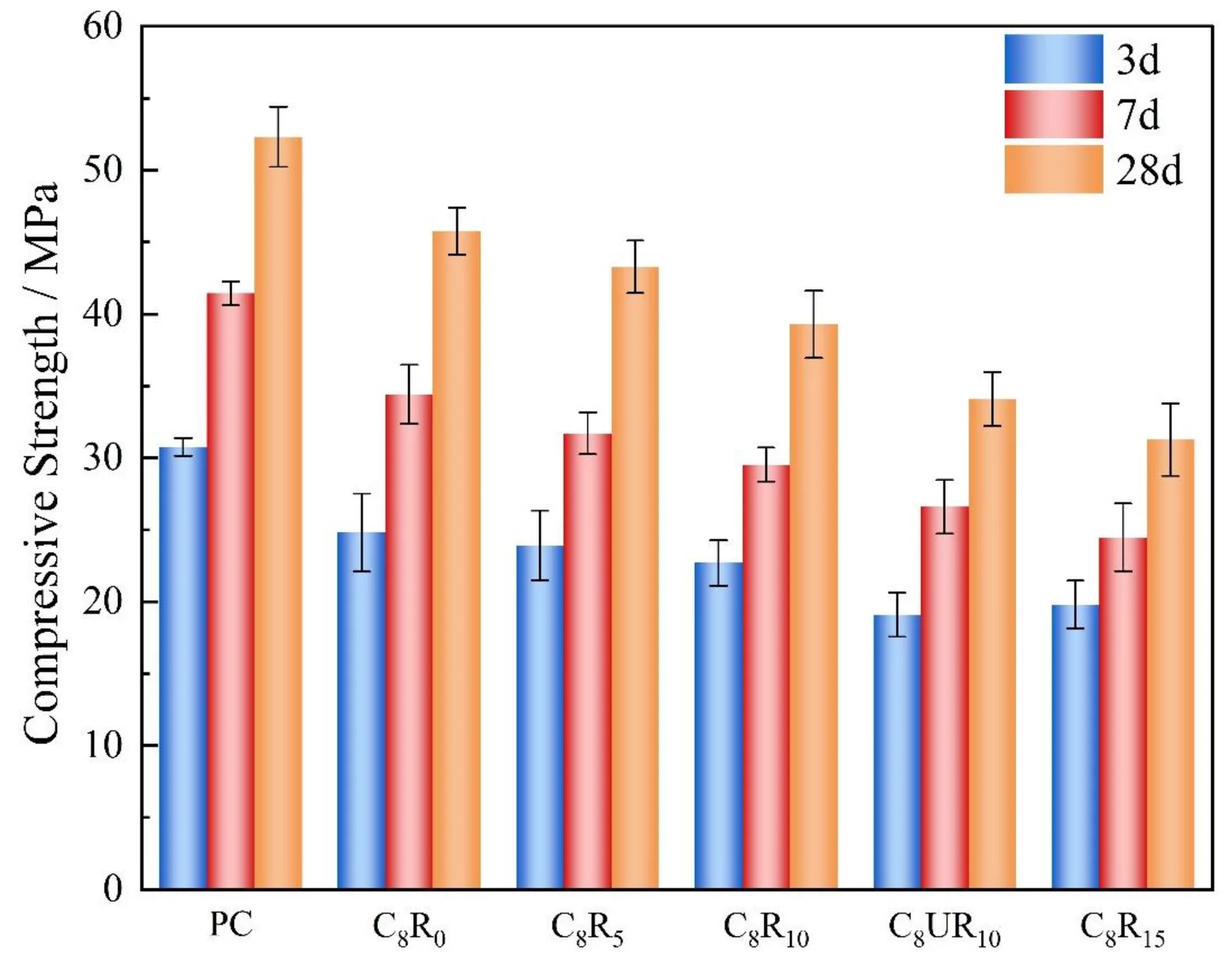
Compressive strength of concrete with different CR contents.

Figure 9 shows that compressive strength decreased continuously with increasing rubber content at a fixed CSA replacement level of 8%. The compressive strengths of C8R0 at 3, 7, and 28 d were 24.80, 34.42, and 45.76 MPa, respectively. Increasing the modified CR content to 5%, 10%, and 15% reduced the 28 d strength to 43.28, 39.28, and 31.26 MPa, corresponding to decreases of 5.4%, 14.2%, and 31.7% relative to C8R0. As CR content increased, a greater proportion of rigid natural aggregate was replaced by low-stiffness rubber particles, weakening the internal load-bearing skeleton. The increasing number of rubber–paste interfaces also intensified the effects of interfacial defects and stress concentrations. Cracks could therefore initiate and propagate more readily around rubber particles, leading to a progressive decline in compressive strength.

At the same CR content of 10%, C8R10 achieved compressive strengths of 22.70, 29.53, and 39.28 MPa at 3, 7, and 28 d, respectively, exceeding the corresponding values of 19.09, 26.59, and 34.09 MPa for the untreated-rubber mixture C8UR10 by 18.9%, 11.1%, and 15.2%. These results indicate that KH570 treatment effectively mitigated the strength loss associated with CR incorporation. The improved mechanical performance is consistent with enhanced interfacial compatibility between the treated rubber particles and the cementitious matrix. Hydrolysis of KH570 produces silanol-containing species, which may modify the surface characteristics and wettability of CR and thereby promote more effective contact with the surrounding cement paste. This interpretation is also consistent with the SEM and FTIR observations discussed in Section 3.4; however, the present results do not provide direct evidence for the formation of specific chemical bonds at the rubber–cement interface. Nevertheless, the compressive strength of C8R10 remained lower than that of C8R0, indicating that KH570 treatment mitigated, but did not fully eliminate, the adverse effects associated with the low stiffness of rubber and the replacement of natural aggregate.

### 3.3. Flexural Strength

**Figure 10:**
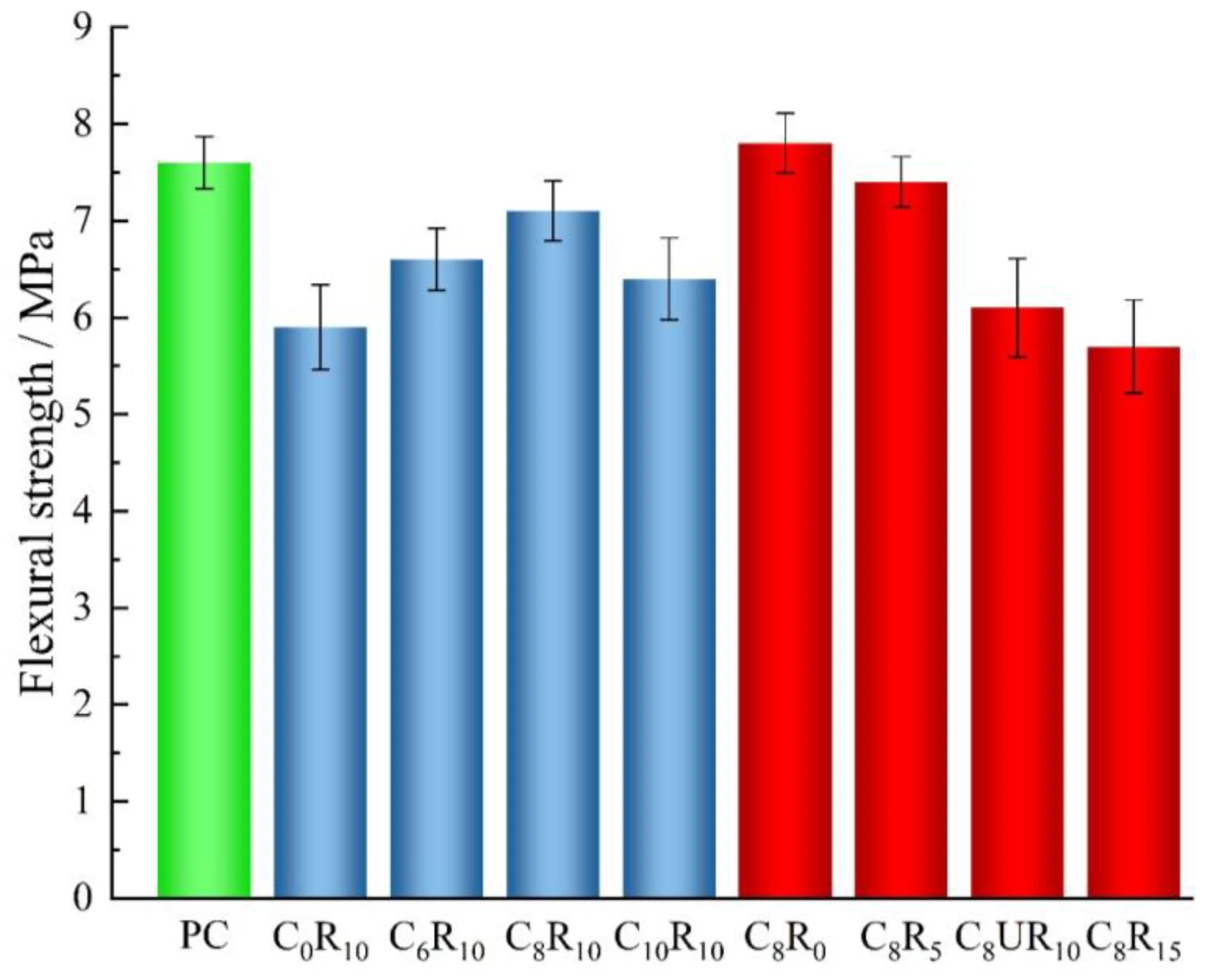
Flexural strength of concrete.

Figure 10 presents the flexural strengths of the concrete mixtures. PC exhibited a flexural strength of 7.6 MPa, whereas C0R10, containing 10% modified rubber, achieved 5.9 MPa, a reduction of 22.4%. Rubber particles have a substantially lower elastic modulus and stiffness than natural sand, so their replacement of river sand weakens the rigid load-bearing skeleton. The hydrophobic rubber surface and its poor compatibility with cement paste also favor pore formation and bonding defects in the ITZ. These defects facilitate crack initiation and propagation under bending loads, reducing flexural strength. At a fixed rubber content of 10%, flexural strength initially increased and then decreased with CSA content. C6R10, C8R10, and C10R10 exhibited flexural strengths of 6.6, 7.1, and 6.4 MPa, respectively, representing increases of 11.9%, 20.3%, and 8.5% relative to C0R10. C8R10 achieved the highest value, indicating that 8% CSA effectively compensated for part of the rubber-induced loss in flexural strength. An appropriate CSA content can improve particle packing and reduce matrix pores through microfilling. The reactive fraction of CSA may also contribute to secondary pozzolanic reactions with Ca(OH)_2_ produced during cement hydration, potentially promoting additional C–S–H formation and improving the compactness of the matrix and ITZ. However, increasing CSA to 10% reduced flexural strength by 9.9% relative to C8R10. This may be attributed to stronger cement dilution and greater mixing water demand arising from the porous structure and high water absorption of excessive CSA, leaving more unreacted particles and local pores. C8R0 achieved a flexural strength of 7.8 MPa, 2.6% higher than PC, indicating that incorporating 8% CSA alone did not adversely affect flexural performance and may instead have contributed to matrix refinement through microfilling and potential pozzolanic reactions.

At a fixed CSA content of 8%, flexural strength decreased progressively with increasing modified rubber content. The flexural strengths of C8R0, C8R5, C8R10, and C8R15 were 7.8, 7.4, 7.1, and 5.7 MPa, respectively. Relative to C8R0, incorporating 5%, 10%, and 15% modified rubber reduced flexural strength by 5.1%, 9.0%, and 26.9%, respectively. Strength losses were relatively limited at low rubber contents but became pronounced at 15%. Increasing rubber content creates more rubber–paste interfaces and further reduces overall stiffness and interfacial stress transfer capacity, enabling cracks in the tensile zone to propagate more readily along interfacial defects. The flexural strength of C8R10 was 7.1 MPa, 16.4% higher than the 6.1 MPa obtained for C8UR10. This indicates that KH570 surface treatment effectively improved the flexural performance of the rubberized concrete, which is consistent with improved interfacial compatibility between the rubber particles and the cement matrix. KH570 treatment may increase rubber surface wettability and promote more effective contact with the surrounding cement paste, thereby reducing interfacial defects and delaying crack initiation and propagation. Nevertheless, the flexural strength of C8R10 remained 9.0% below that of C8R0, demonstrating that interfacial modification mitigated, but did not fully eliminate, the adverse effects of low rubber stiffness and aggregate replacement.

### 3.4. Microstructure

#### 3.4.1. XRD

**Figure 11:**
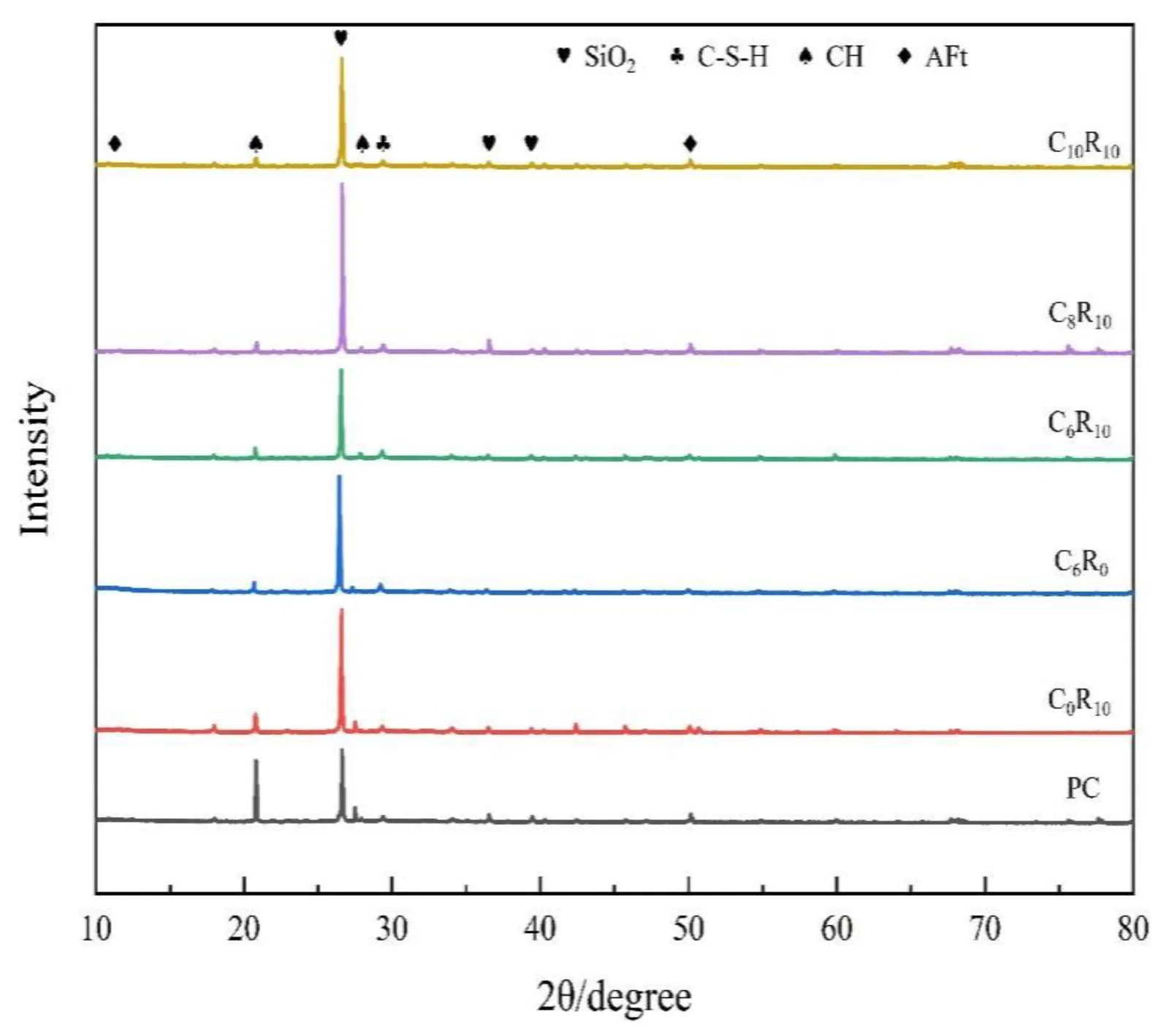
XRD patterns of concrete after 28 d of curing.

Figure 11 shows the XRD patterns of the concrete mixtures after 28 d of curing. The specimens exhibited broadly similar major phases, with diffraction features associated with SiO_2_, Ca(OH)_2_ (CH), ettringite (AFt), and calcium silicate hydrate (C–S–H), and no obvious new crystalline phases appeared among the mixtures. This indicates that CSA and CR primarily affected the extent of existing hydration reactions and the relative composition of hydration products without substantially changing their basic types. This result is generally consistent with the findings of Zhang et al. [25]. The diffraction peak positions of PC and C0R10 were similar, indicating that replacing part of the sand with CR did not generate new crystalline phases. Rubber is an organic polymer and generally does not participate in the formation of inorganic mineral phases during cement hydration; its effects are therefore mainly associated with the rubber–matrix ITZ and pore structure rather than changes in hydration product types. After CSA incorporation, C6R0, C6R10, C8R10, and C10R10 exhibited prominent SiO_2_ diffraction features near 2θ = 26.6°. The weakening of CH-related diffraction features after CSA incorporation is consistent with a possible pozzolanic contribution of CSA. However, the extent of CH consumption and additional C–S–H formation cannot be quantitatively determined from the present XRD results alone. Comparing C6R0 and C6R10 shows that rubber addition at the same CSA content did not noticeably change the principal peak positions, further indicating that rubber did not alter the main crystalline hydration products. Differences in peak intensity among C6R10, C8R10, and C10R10 indicate that CSA content affected the degree of hydration. Increasing CSA from 6% to a suitable level allowed microfilling and pozzolanic reactions to jointly densify the matrix; C8R10 can be considered to have undergone a comparatively more extensive secondary reaction and to possess a more favorable hydration product composition. Further increasing CSA to 10% reduced clinker content and limited the CH available for secondary reactions, potentially leaving some SiO_2_ as unreacted particles. Excessive CSA therefore did not necessarily further increase hydration, which can also explain the initial improvement and subsequent decline in mechanical performance with increasing CSA content.

#### 3.4.2. SEM Observations

**Figure 12:**
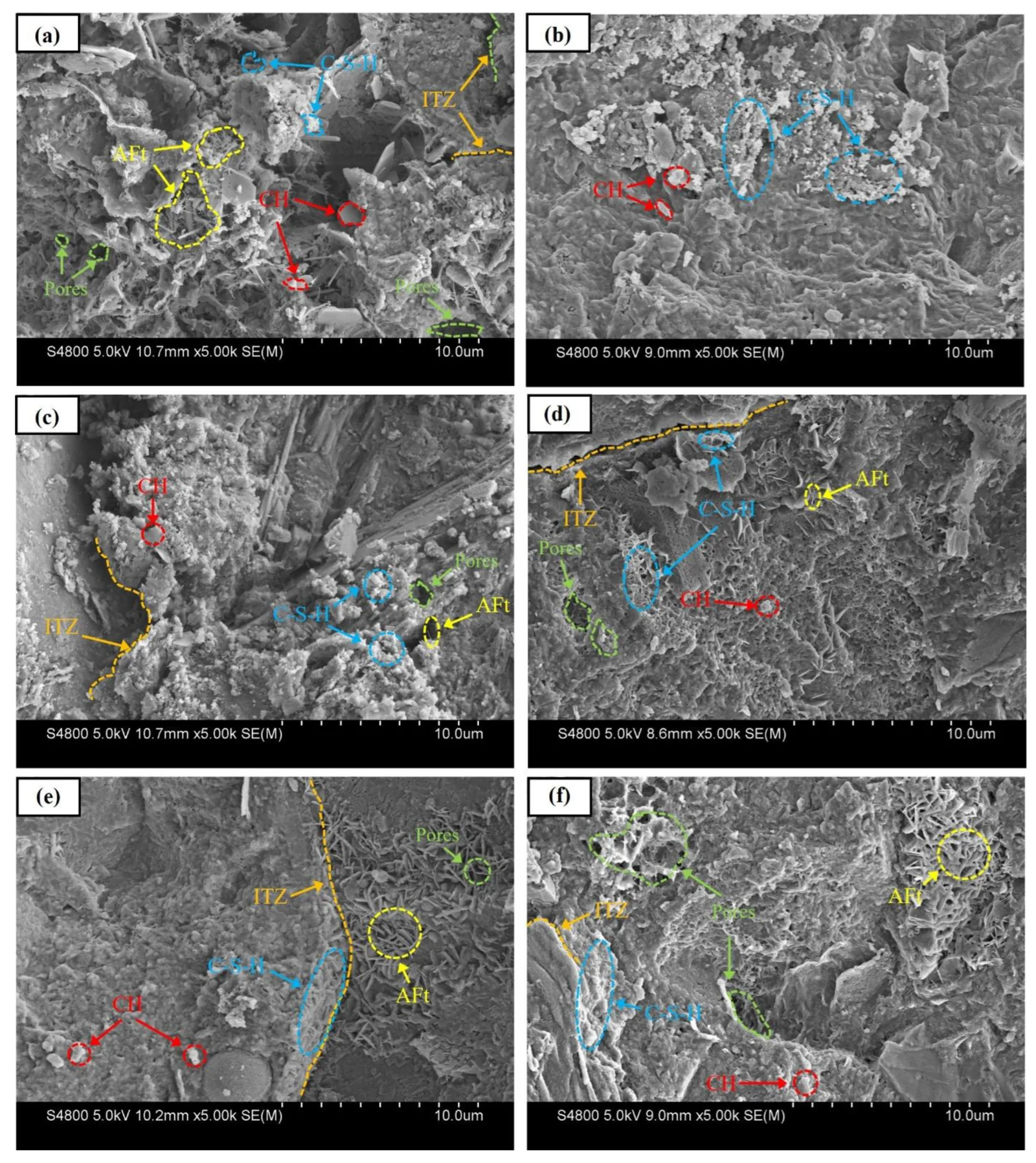
SEM images of (**a**) PC, (**b**) C8R0, (**c**) C8UR10, (**d**) C8R10, (**e**) C6R10, and (**f**) C10R10.

Figure 12a–f shows the microstructures of PC, C8R0, C8UR10, C8R10, C6R10, and C10R10, respectively. In Figure 12a, the PC matrix contains distinct plate-like CH, needle- or rod-like AFt, and flocculent or gel-like C–S–H, together with pores and locally loose regions. Some AFt occurs as needle bundles within pores, and connections between hydration products are not fully continuous, indicating residual microstructural defects in the plain concrete matrix.

Compared with PC, C8R0 in Figure 12b exhibits a more continuous matrix, with widely distributed flocculent and clustered C–S–H and fewer conspicuous CH plates. This is mainly associated with the participation of reactive SiO_2_ in the pozzolanic reaction, which consumes hydration-derived Ca(OH)_2_ and produces secondary C–S–H gel. Fine CSA particles further improve the pore structure through filling. Incorporating 8% CSA thus promoted hydration product redistribution and matrix densification, consistent with the improvement in mechanical performance.

Figure 12c shows the specimen containing CR without KH570 treatment, designated C8UR10. Although C–S–H and small amounts of AFt and CH remain visible, a distinct ITZ occurs between CR and the cement matrix, accompanied locally by pores and loose hydration products. The strong hydrophobicity and low surface activity of untreated rubber impair wetting and bonding with cement paste, hindering the formation of a continuous, dense hydration product layer on the CR surface and producing a relatively weak ITZ. Thus, although 8% CSA can improve the cement matrix through pozzolanic reactions, interfacial defects associated with untreated CR may still limit mechanical performance.

In contrast, KH570-treated C8R10 in Figure 12d exhibits a more continuous hydration product structure. The ITZ surrounding CR is denser, with abundant C–S–H gel at the interface and needle- or rod-like AFt interwoven with surrounding gel-like hydration products. KH570 treatment improves compatibility between the CR surface and cement paste, enabling hydration products to adhere more effectively to rubber and reducing interfacial pores and debonding defects. Meanwhile, secondary C–S–H associated with 8% CSA further fills micropores in the ITZ and matrix, producing a more continuous interface. The synergistic effects of CSA pozzolanic reactions and KH570-induced CR surface modification therefore contribute substantially to the improved microstructure of C8R10.

In C6R10, shown in Figure 12e, a prominent accumulation of needle- or rod-like AFt is visible on the right, while C–S–H gel is distributed in the central and left regions. Local pores and relatively loose hydration products remain apparent. At 6% CSA, pozzolanic reactions and microfilling already improve the matrix and ITZ, but the relatively limited supply of reactive siliceous constituents means that secondary C–S–H does not optimally fill pores and interfacial defects. The overall microstructural uniformity and compactness therefore remain below those of C8R10.

When CSA content increases to 10%, as shown in Figure 12f, C–S–H and local needle- or rod-like AFt remain visible, but hydration products are distributed less uniformly, with conspicuous central pores and locally loose regions. Excessive cement replacement by CSA reduces the effective clinker mineral content. As pozzolanic reactions proceed, the Ca(OH)_2_ available for further reaction also becomes limited, preventing some CSA particles from fully participating in secondary reactions. Increasing CSA from 8% to 10% therefore does not produce further uniform densification; instead, local defects persist, explaining why mechanical performance does not continue to improve beyond the optimum dosage.

#### 3.4.3. FTIR

**Figure 13:**
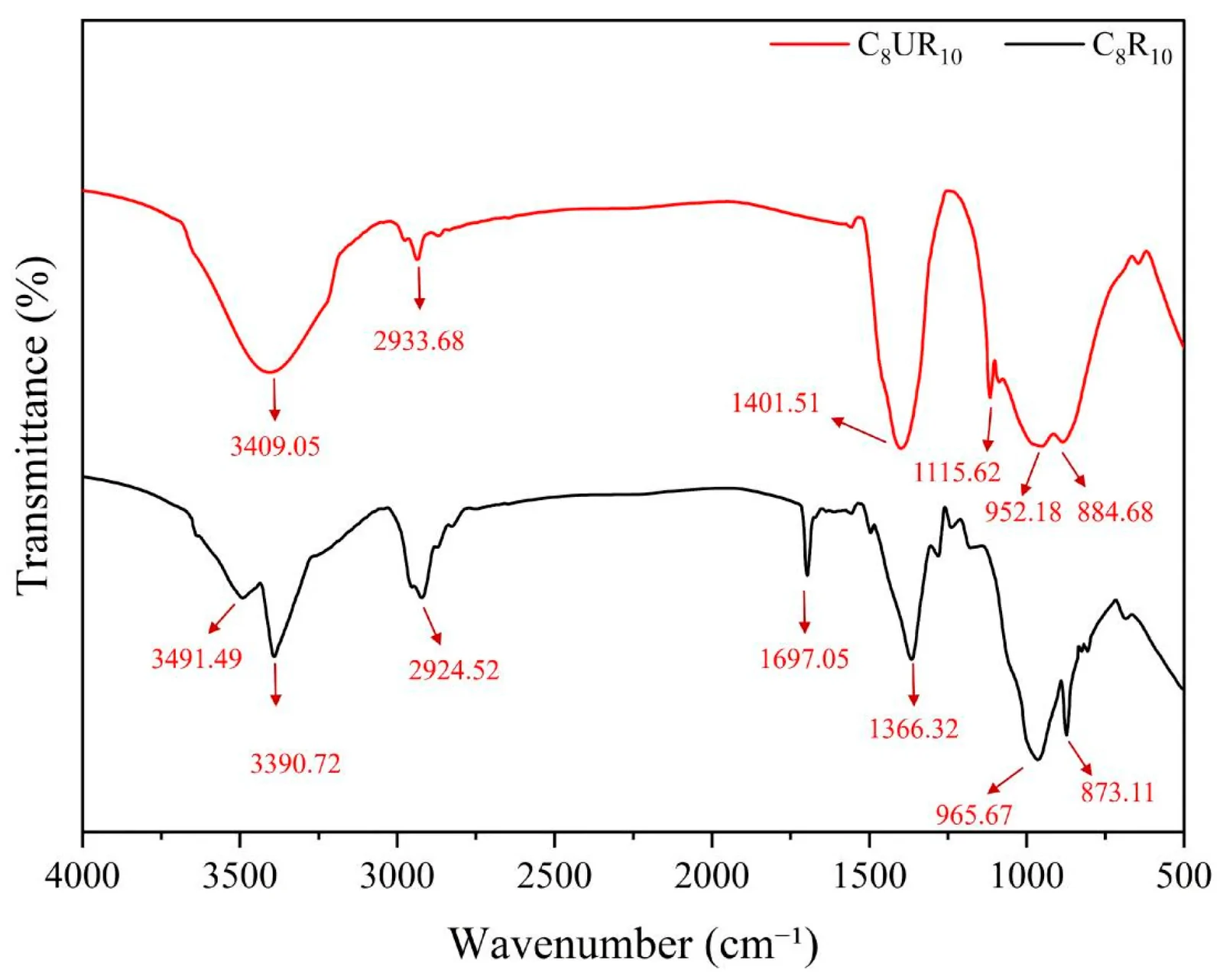
FTIR spectra of corn straw ash concrete containing untreated and KH570-modified crumb rubber.

Figure 13 presents the FTIR spectra of the untreated-rubber mixture C8UR10 and the KH570-modified rubber mixture C8R10. The main absorption bands occur at broadly similar positions, indicating that KH570 treatment did not substantially change the principal hydration product types. However, shifts, splitting, and intensity changes in some characteristic bands indicate changes in the chemical environment of the rubber surface and its interactions with cement hydration products. C8UR10 exhibits a broad absorption band near 3409.05 cm^−1^, mainly associated with O–H stretching in hydration products and adsorbed water. Following KH570 modification, C8R10 exhibits two absorption peaks near 3491.49 and 3390.72 cm^−1^, indicating a changed chemical environment for hydroxyl groups. Absorption peaks near 2933.68 cm^−1^ for C8UR10 and 2924.52 cm^−1^ for C8R10 can be assigned to –CH_2_– stretching in rubber molecular chains. The shift to a lower wavenumber after treatment indicates some physical entanglement or interfacial interaction between KH570 organic functional groups and rubber chains, without substantial disruption of the original organic backbone. C8R10 also exhibits a new absorption feature near 1697.05 cm^−1^. This band may include a contribution from C=O vibrations in the methacrylate group of KH570 and may partially overlap with bending vibrations of bound water. This change indicates that KH570 molecules or their hydrolysis–condensation products were retained in the rubber–cement interfacial region. At lower wavenumbers, the C8UR10 peaks near 1401.51, 952.18, and 884.68 cm^−1^ shift to approximately 1366.32, 965.67, and 873.11 cm^−1^ in C8R10, respectively. Bands near 1400 and 870–890 cm^−1^ are mainly associated with CO_3_^2−^ vibrations, whereas the band near 950–970 cm^−1^ primarily corresponds to Si–O stretching in C–S–H gel. These shifts indicate that KH570 treatment changed the local chemical environments of silicate structures and carbonate groups and may have strengthened interactions between rubber particles and cement hydration products. The C8UR10 absorption band near 1115.62 cm^−1^ may be associated with SO_4_^2−^ vibrations, with a possible overlapping contribution from Si–O–Si vibrations. Changes in band shape in this region after treatment may be related to siloxane structures formed after KH570 hydrolysis and their interactions with hydration products.

### 3.5. Model Prediction Results

**Figure 14:**
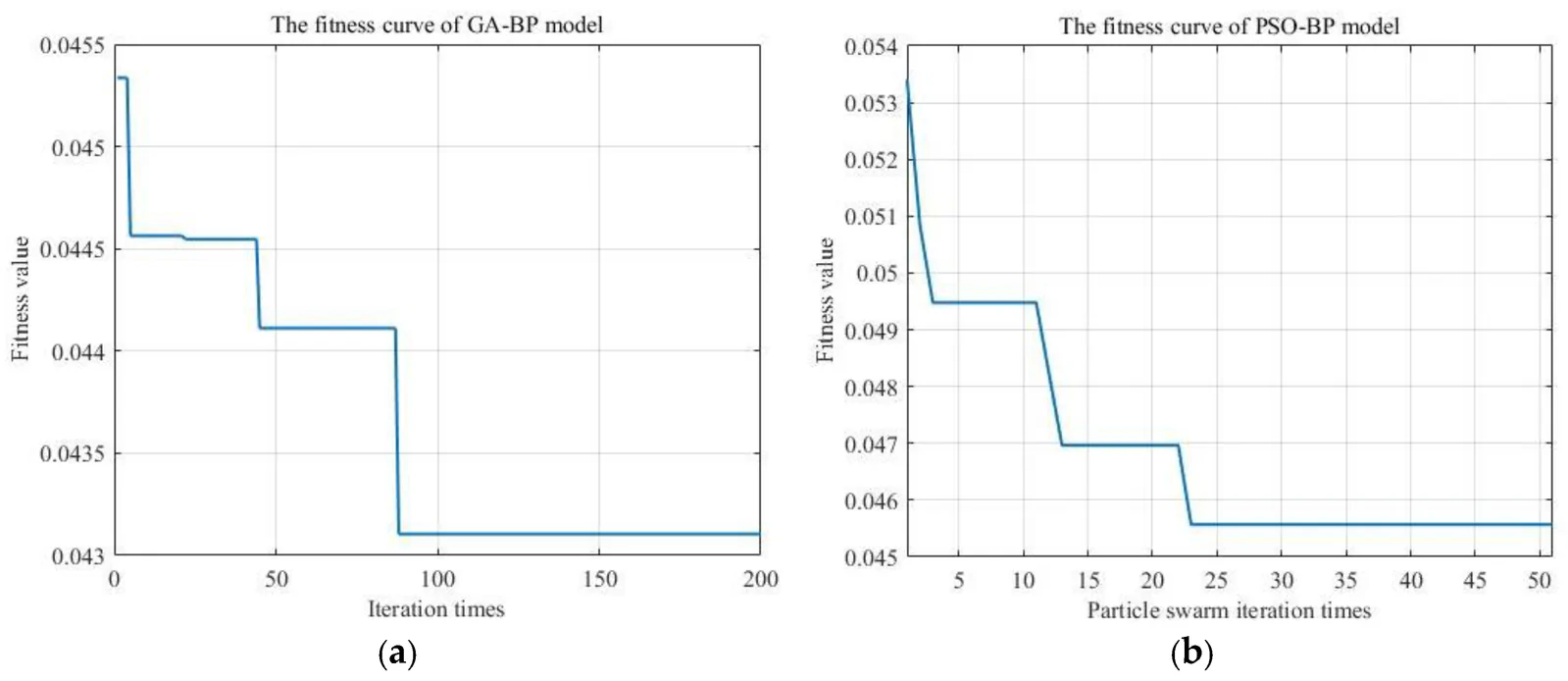
Convergence curves of (**a**) GA-BP and (**b**) PSO-BP.

Figure 14 illustrates the evolution of fitness during GA-BP and PSO-BP optimization. For both models, fitness decreased stepwise with increasing iteration number and eventually stabilized, indicating that GA and PSO effectively searched for improved initial weights and biases for the BP network. For GA-BP, fitness decreased rapidly from an initial value of 0.0453 to 0.0446 during the early iterations, followed by several short plateaus. More pronounced decreases occurred at iterations 45 and 88, after which fitness stabilized at approximately 0.0431 and remained essentially unchanged through iteration 200. This indicates that selection, crossover, and mutation progressively moved the genetic algorithm toward a better solution, with convergence largely achieved after approximately 90 iterations. The extended plateaus indicate that some stages mainly involved local search, with further improvement occurring only when individuals with better fitness emerged. By comparison, PSO-BP fitness fell rapidly from 0.0535 to 0.0495 during the first few iterations, decreased further at iterations 12 and 23, and finally stabilized at 0.0455. The curve remained essentially unchanged after iteration 23, indicating that PSO used individual and global best positions to rapidly adjust the search direction, achieving faster initial optimization and convergence.

Table 4 summarizes model performance on the training and test sets. BP achieved RMSE values of 2.6226 and 2.8515 MPa and corresponding R values of 0.95216 and 0.94051, respectively. The conventional BP network thus captured the nonlinear relationship between the inputs and compressive strength to some extent, although prediction errors remained. GA optimization reduced training and test RMSE to 2.2617 and 2.5186 MPa, respectively, representing reductions of 13.76% and 11.67% relative to BP. The corresponding R values increased to 0.96594 and 0.94813, indicating that optimizing initial weights and biases improved the fit. PSO-BP achieved training and test RMSE values of 1.9322 and 2.1713 MPa, 26.33% and 23.85% lower than BP, with R values of 0.98313 and 0.96256, respectively. Its test RMSE was also 13.79% lower than that of GA-BP, indicating that PSO-BP combined lower prediction error with a stronger predicted–experimental correlation under the data split and parameter settings used here.

**Figure 15:**
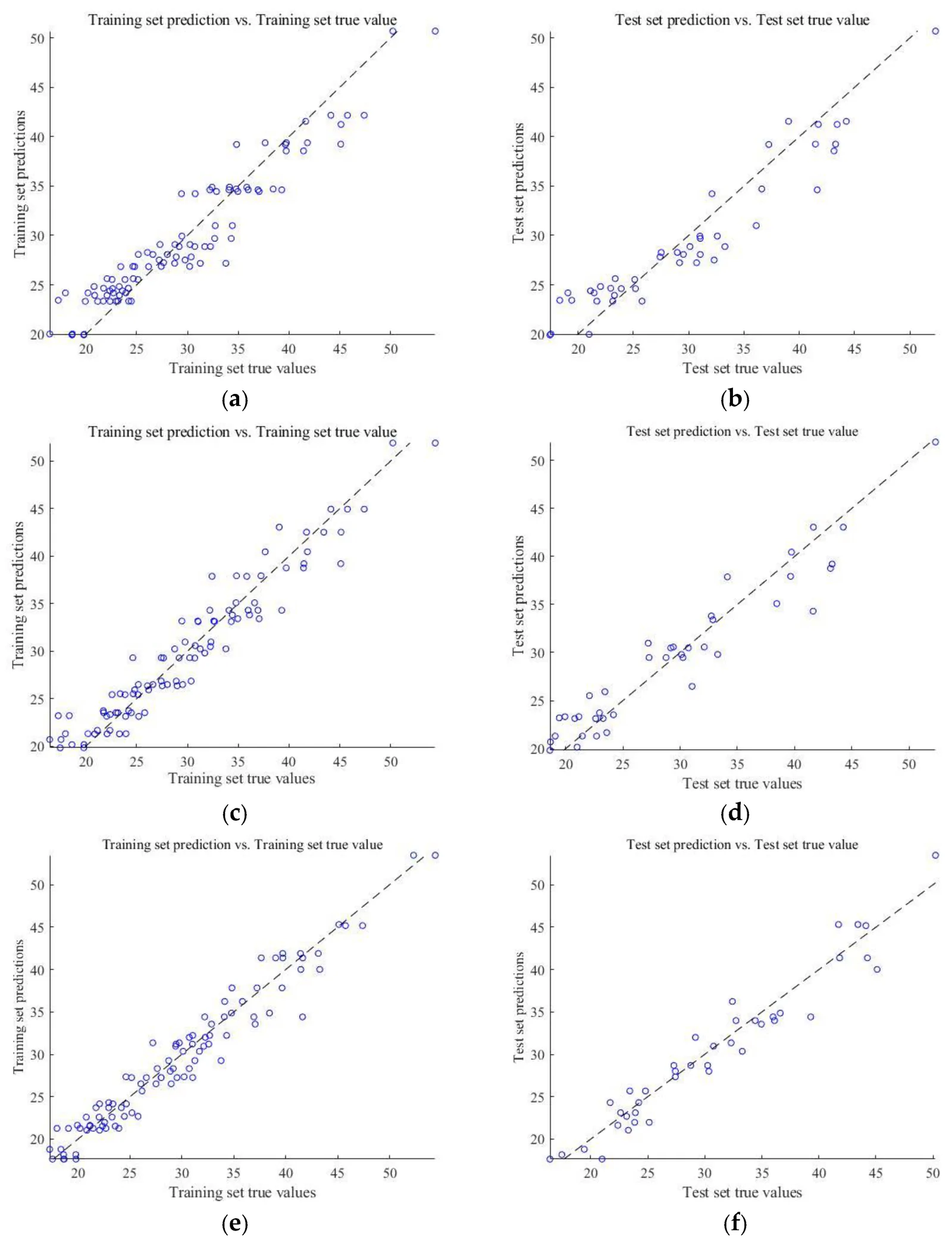
Regression plots for the three neural networks: (**a**) BP training set; (**b**) BP test set; (**c**) GA-BP training set; (**d**) GA-BP test set; (**e**) PSO-BP training set; and (**f**) PSO-BP test set.

The predicted-versus-experimental scatter plots in Figure 15 further illustrate model fit. All three models captured the overall variation in compressive strength, but some BP predictions deviated noticeably from the line of equality, indicating an insufficient fit for samples at different strength levels. GA and PSO optimization generally improved agreement between predicted and experimental values. PSO-BP exhibited a stronger linear correlation, consistent with the R values in Table 4. However, correlation primarily measures agreement in trends; a high R does not exclude systematic prediction bias and must therefore be considered alongside RMSE. The highest R and lowest RMSE achieved by PSO-BP support its superior overall predictive performance in this comparison.

**Figure 16:**
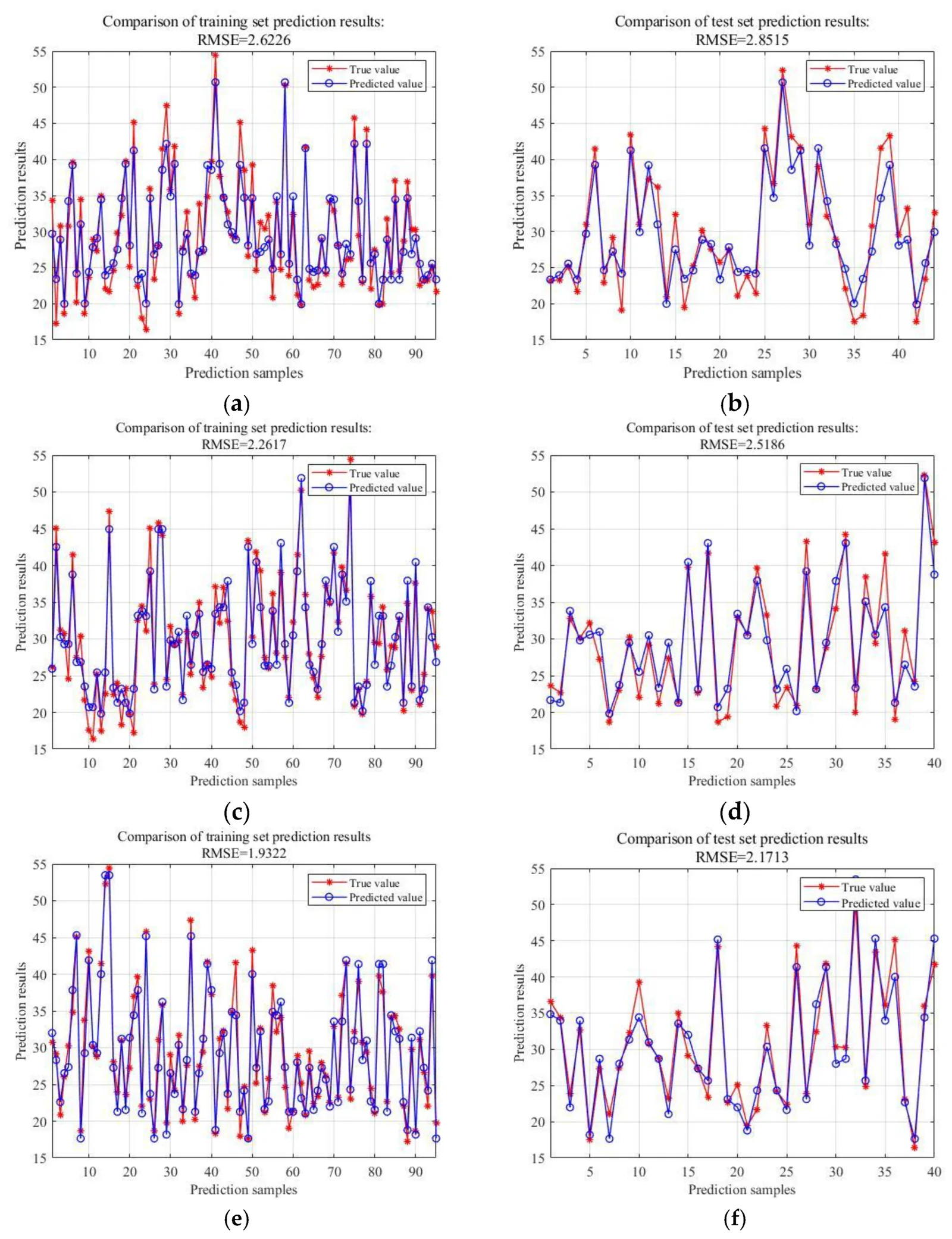
Sample-by-sample predictions of the three neural networks: (**a**) BP training set; (**b**) BP test set; (**c**) GA-BP training set; (**d**) GA-BP test set; (**e**) PSO-BP training set; and (**f**) PSO-BP test set.

Figure 16 compares sample-by-sample predictions for the training and test sets. The prediction curves of all three models followed the main fluctuations in the experimental values, indicating that the selected mix variables and curing age provided useful information for compressive strength prediction. BP exhibited noticeable deviations for some samples with high or low strengths. Parameter optimization improved the overall agreement between predicted and experimental curves. PSO-BP achieved the lowest overall error, although some samples remained overestimated or underestimated, demonstrating that optimization reduced aggregate prediction error without eliminating all individual errors. These residual deviations may reflect experimental variability, the limited sample size, and input variables that do not fully represent differences in materials and preparation procedures. For all three models, test RMSE exceeded training RMSE and test R was lower, indicating some performance loss on previously unseen samples. Nevertheless, PSO-BP retained the lowest RMSE and highest R on the test set, preserving its accuracy advantage in this evaluation. The average error rates in Table 2 showed the same trend: the test values for BP, GA-BP, and PSO-BP were 5.13, 4.52, and 2.35, respectively, further supporting the lower overall prediction error of PSO-BP. These findings are consistent with population-based optimization improving network initialization and subsequent training performance.

To complement the record-wise random-split results presented above, five-fold mixture-level cross-validation was performed following the procedure described in Section 2.4. This evaluation assessed prediction performance for mixtures whose observations were entirely excluded from model fitting. Table 5 summarizes the MAE, RMSE, and R^2^ values obtained for the held-out mixtures, reported as the mean and sample standard deviation across the five folds.

Table 5 summarizes the prediction performance of the three models under five-fold mixture-level cross-validation. PSO-BP achieved the lowest mean MAE and RMSE of 2.32 and 3.15 MPa, respectively, together with the highest mean R^2^ of 0.87. Compared with BP, its mean MAE and RMSE decreased by 18.0% and 23.7%, respectively; the corresponding reductions relative to GA-BP were 6.5% and 12.7%. These results indicate that PSO-BP provided the best average prediction accuracy for the held-out mixtures under the adopted evaluation settings. The standard deviations reveal variation in predictive performance across the held-out mixture groups. PSO-BP exhibited the smallest fold-to-fold variation in MAE and R^2^, whereas its RMSE standard deviation was similar to that of GA-BP. Thus, its advantage was primarily reflected in lower average prediction errors rather than uniformly lower variability across all metrics.

## 4. Conclusions

This study evaluated CSA incorporation and KH570-treated CR in concrete through strength testing and complementary microstructural observations. The following conclusions are drawn within the investigated material and mixture ranges:

(1) At 10% CR replacement, 8% CSA was the highest-performing CSA dosage among the investigated levels. C8R10 reached 28 d compressive and flexural strengths of 39.28 and 7.1 MPa, respectively, exceeding C0R10 by 15.1% and 20.3%. This identifies a favorable tested composition rather than a universal optimum.
(2) At matched CSA and CR contents, KH570-treated C8R10 exceeded untreated C8UR10 in 28 d compressive and flexural strengths by 15.2% and 16.4%, respectively. Nevertheless, increasing CR from 0% to 15% at 8% CSA reduced these strengths by 31.7% and 26.9%. The combined approach therefore mitigated, but did not eliminate, rubber-related strength losses.
(3) XRD, SEM, and FTIR observations were consistent with matrix refinement and improved rubber–paste interfacial compatibility, supporting their possible contributions to the strength improvements achieved through CSA incorporation and KH570 treatment.
(4) PSO-BP achieved the best average performance under five-fold mixture-level cross-validation, with an MAE of 2.32 MPa, an RMSE of 3.15 MPa, and an R^2^ of 0.87, indicating its potential for strength prediction within the investigated mixture range.

Future research should focus on quantitative characterization of pore structure and rubber–paste interfacial properties, systematic durability evaluation, and independent validation of the prediction models to further assess the engineering potential of CSA–CR concrete.

## Figures and Tables

**Table 1:** Comparison with related studies and the scope of the present investigation.

Study	Materials and Modification	Connection to the Present Study
Yu et al. (2021) [24]	Rubber–cement composites; comparative silane treatments, including KH570 and KH550.	Provides the basis for selecting KH570; The present study examines its use in a CSA-containing matrix.
Shao et al. (2022) [22]	Recycled tire rubber concrete incorporating carbon nanotubes (CNTs).	Represents matrix reinforcement with CNTs; The present study combines agricultural ash with rubber surface treatment.
Shang et al. (2024) [11]	Processed corn straw ash used as a supplementary cementitious material in mortar.	Establishes the relevance of CSA processing and binder substitution to the matrix component of the present system.
Hassanean et al. (2024) [27]	Rice husk ash (RHA) as cement replacement and CR as fine-aggregate replacement in reinforced concrete beams.	Examines an agricultural ash–rubber system at beam scale; The present study addresses CSA and KH570 treatment at material scale.
Ji and Xia (2025) [8]	Unburned straw powder and recycled rubber, with KH570 modification.	Provides a related KH570–agricultural-residue system; The present work uses combustion-derived CSA as cement replacement.
Zhang et al. (2025) [25]	Two-stage-combusted CSA and KH550-treated CR as cement and sand replacements.	Closest combined-constituent study. The present work evaluates CSA–KH570 mixtures and a matched treated/untreated comparison.
Hussein et al. (2025) [26]	RHA levels of 0%, 10%, and 20%; CR levels of 0%, 5%, and 10% in reinforced concrete beams.	Connects ash–rubber composition with structural response. The present work links CSA dosage and treatment with material strength and microstructure.
Han et al. (2026) [28]	Concrete containing untreated and KH570-pretreated CR.	Extends evidence on KH570-treated rubber to fatigue; The present study concentrates on CSA-containing concrete and static strength.

**Table 2:** Concrete mix proportions (kg/m^3^).

Mixtures	Water	Cement	Sand	Aggregate (4.75 mm~9.5 mm)	Coarse Aggregate (9.5 mm~19 mm)	CR	CSA	Water Reducer
PC	195	487	613	340	794	0	0	0.49
C0R10	195	487	491	340	794	89.46	0	0.49
C6R10	195	457.78	491	340	794	89.46	29.22	0.49
C8R10	195	448.04	491	340	794	89.46	38.96	0.49
C8UR10	195	448.04	491	340	794	89.46	38.96	0.49
C10R10	195	438.3	491	340	794	89.46	48.7	0.49
C6R0	195	457.78	613	340	794	0	29.22	0.49
C6R5	195	457.78	552	340	794	44.73	29.22	0.49
C6R15	195	457.78	429	340	794	134.19	29.22	0.49
C8R0	195	448.04	613	340	794	0	38.96	0.49
C8R5	195	448.04	552	340	794	44.73	38.96	0.49
C8R15	195	448.04	429	340	794	134.19	38.96	0.49
C10R0	195	438.3	613	340	794	0	48.7	0.49
C10R5	195	438.3	552	340	794	44.73	48.7	0.49
C10R15	195	438.3	429	340	794	134.19	48.7	0.49

**Table 3:** Chemical compositions of CSA (wt%).

SiO_2_	Al_2_O_3_	K_2_O	CaO	MgO	P_2_O_5_	SO_3_	Fe_2_O_3_	Na_2_O	Other	LOI
59.42	11.53	5.21	3.85	3.3	1.82	1.2	2.8	1.4	0.25	3.1

**Table 4:** Evaluation metrics for the training and test sets of the three models.

Evaluation	BP	BP	PSO-BP	PSO-BP	GA-BP	GA-BP
Evaluation	Training Set	Test Set	Training Set	Test Set	Training Set	Test Set
MAE	1.6756	1.8712	1.4857	1.7332	1.5599	1.7938
RMSE	2.6226	2.8515	1.9322	2.1713	2.2617	2.5186
R^2^	0.8601	0.85374	0.91769	0.90197	0.89104	0.88575
R	0.95216	0.94051	0.98313	0.96256	0.96594	0.94813
Average error rate	3.74	5.13	1.77	2.35	2.68	4.52

**Table 5:** Performance of BP, GA-BP, and PSO-BP under five-fold mixture-level cross-validation.

Model	MAE (MPa)	RMSE (MPa)	R^2^
BP	2.83 ± 0.85	4.13 ± 1.05	0.82 ± 0.09
PSO-BP	2.32 ± 0.55	3.15 ± 0.86	0.87 ± 0.03
GA-BP	2.48 ± 0.63	3.61 ± 0.85	0.84 ± 0.08

## Data Availability

The datasets used and/or analyzed during the current study are available from the corresponding author upon reasonable request due to privacy.
